# Current Knowledge about Gastric Microbiota with Special Emphasis on *Helicobacter pylori-*Related Gastric Conditions

**DOI:** 10.3390/cimb46050299

**Published:** 2024-05-20

**Authors:** Luigi Santacroce, Skender Topi, Lucrezia Bottalico, Ioannis Alexandros Charitos, Emilio Jirillo

**Affiliations:** 1 Section of Microbiology and Virology, Interdisciplinary Department of Medicine, School of Medicine, University of Bari ‘Aldo Moro’, 70124 Bari, Italy; emilio.jirillo@uniba.it; 2 Department of Clinical Disciplines, University ‘Alexander Xhuvani’ of Elbasan, 3001 Elbasan, Albania; skender.topi@uniel.edu.al (S.T.);; 3 Istituti Clinici Scientifici Maugeri IRCCS, Pneumology and Respiratory Rehabilitation Unit, Institute of Bari, 70124 Bari, Italy; alexanestesia@hotmail.com

**Keywords:** gastric microbiota, *Helicobacter pylori*, dysbiosis, cancer, immunity, inflammation

## Abstract

The gastric milieu, because of its very low acidic pH, is very harsh for bacterial growth. The discovery of *Helicobacter pylori* (*H.p.*) has opened a new avenue for studies on the gastric microbiota, thus indicating that the stomach is not a sterile environment. Nowadays, new technologies of bacterial identification have demonstrated the existence of other microorganisms in the gastric habitat, which play an important role in health and disease. This bacterium possesses an arsenal of compounds which enable its survival but, at the same time, damage the gastric mucosa. Toxins, such as cytotoxin-associated gene A, vacuolar cytotoxin A, lipopolysaccharides, and adhesins, determine an inflammatory status of the gastric mucosa which may become chronic, ultimately leading to a gastric carcinoma. In the initial stage, *H.p.* persistence alters the gastric microbiota with a condition of dysbiosis, predisposing to inflammation. Probiotics and prebiotics exhibit beneficial effects on *H.p.* infection, and, among them, anti-inflammatory, antioxidant, and antibacterial activities are the major ones. Moreover, the association of probiotics with prebiotics (synbiotics) to conventional anti-*H.p.* therapy contributes to a more efficacious eradication of the bacterium. Also, polyphenols, largely present in the vegetal kingdom, have been demonstrated to alleviate *H.p.-*dependent pathologies, even including the inhibition of tumorigenesis. The gastric microbiota composition in health and disease is described. Then, cellular and molecular mechanisms of *H.p.-*mediated damage are clarified. Finally, the use of probiotics, prebiotics, and polyphenols in experimental models and in patients infected with *H.p.* is discussed.

## 1. Introduction

*Helicobacter* (*H.*) *pylori* (*p.*) is a spiralized, microaerophilic Gram-negative bacterium showing a typical corkscrew motility thanks to a tuft of polar flagella. It has developed an effective molecular mechanism to resist the acidic pH of the stomach, as well as several virulence factors to facilitate bacterial tolerance in the acidic microenvironment such as urease, arginase, sialic-acid-binding adhesin (SABA), duodenal ulcer promoter gene A, outer membrane protein Q, and outer membrane protein Z, all useful in colonizing the mucosa [1,2]. Although the infection is ubiquitous and affects several animals and humans, the contagion dynamics are not fully understood. A consensus has been established on the inter-individual transmission via oral (mainly in familiars, especially from mothers and siblings) or fecal–oral routes, as well as possible infection through the consumption of drinks or foods handled in poor hygiene conditions. Usually, infected individuals remain asymptomatic but persistent infection may lead to chronic gastritis, peptic ulcer, gastric cancer, and mucosa-associated lymphoid tissue lymphoma (MALToma) [3,4,5].

*H.p.* pathogenesis and colonization depend on multiple factors, such as urease, adhesins, outer membrane proteins, neutrophil-activating protein A, cytotoxin-associated gene A (Cag A), vacuolar cytotoxin A (Vac A), and the type IV secretion system (T4SS) [1].

In particular, several studies showed that during the initial phase, *H.p.* utilizes urease for stomach colonization and macrophage interaction. Adherence of bacteria is facilitated by adhesins, such as BabA, SABA, alpA, OipA, and other Hop/Hom families. Then, CagA and VacA translocate into epithelial cells through the T4SS, promoting inflammation, immune suppression, and apoptosis. Finally, gastric cancer risk is associated with a CagA-rich population in comparison with a control population [1,6].

Molecular technologies, such as whole-genome 16S ribosomal RNA sequencing and metagenomics, transcriptomics, proteomics, and metabolomics have contributed to a better characterization of the gastric microbiota composition [7]. Then, in addition to *H.p.*, *Pseudomonadota*, *Bacteroidota*, *Actinomycetota*, and *Fusobacteriota* have been found to be the most prevalent phyla in the gastric mucosa [8]. However, *H.p.* infection accounts for the alteration of the gastric microbiota composition, the so-called dysbiosis. For instance, hypochlorhydria *H.p.+* patients exhibit in their stomach several urease-positive bacteria, e.g., *Actinomyces*, *Corynebacterium*, *Haemophylus*, *Streptococcus*, and *Staphylococcus* [9]. In patients with chronic gastritis, *H.p.*, *Neisseria*, *Prevotella*, and *Streptococcus* were the most prevalent [10]. On the other hand, in gastric carcinoma, *H.p.* decreased, while *Lactobacillaceae* family and the genera *Achromobacter*, *Citrobacter*, *Clostridium*, *Rhodococcus* increased.

*H.p.* and non-*H.p.* microorganisms can cause damage to the gastric mucosa, ranging from inflammation of different degrees and durations to gastric carcinoma. In this respect, *H.p.* mostly exhibits an arsenal of compounds which enable it to bind gastric epithelial cells. CagA, VacA, adhesins, and lipopolysaccharides (LPSs) or endotoxins are major molecules able to cause an inflammatory status of the gastric mucosa, which may transit to a neoplastic condition, such as gastric carcinoma [11]. It is noteworthy that the LPS of *H.p.* shows a phase variation and contains carbohydrate determinants similar to the human Lewis system antigens expressed on the surface of red blood cells [1]. Moreover, despite the presence of the innate lymphoid cells (ILCs) in the gastric mucosa, *H.p.* has been demonstrated to escape immunosurveillance mechanisms, thus allowing its long survival in the stomach. Furthermore, *H. pylori* is able to modify the gastric microbiota, thus causing a condition of dysbiosis, which may generate various diseases in addition to compromising human nutrition [12].

A recent systematic review and meta-analysis reported the global incidence of *H.p.* infection and gastric cancer worldwide and for individual countries, highlighting a progressive decline in this infection in the Western world during the last decade [13]. Probiotics are living microorganisms that benefit the host, when administered in adequate amounts. There is evidence that *Lactobacillaceae* family and *Bifidobacteria* can protect the host against *H.p.* infection through pathogen inhibition, the production of metabolites and enzymes, and the modulation of the immune response [14]. Also, prebiotics, non-digestible oligosaccharides, have the potential to protect the host against *H.p.*, normalizing the equilibrium of the gastric microbiota [15].

Polyphenols, largely contained in the vegetal kingdom (fruits, vegetables, grains, red wine, and extra virgin olive oil), display potent anti-inflammatory and antioxidant activities [16,17]. These natural compounds possess protective activities against *H.p.* infection, mediating a direct antibacterial activity, while reducing the release of proinflammatory cytokines [18].

Specific aims of the current review are the description of the gastric microbiota composition in health and disease, with special reference to *H.p.* infection. *H.p.-*mediated dysbiosis and evolution towards chronic inflammation and cancer are discussed. Finally, new therapeutic options with the administration of probiotics, prebiotics, and polyphenols in order to attenuate *H.p.* pathological consequences are illustrated.

## 2. Gastric Microbiota Composition in Health and Disease

The stomach, in view of its strong acidity, represents a very harsh environment to indigenous bacteria growth. In fact, using traditional culturing methods, bacterial numbers in the gastric mucosa are very scarce (10^3^ colony-forming units per g or mL) [19]. Conversely, adopting new technologies, e.g., DNA sequencing with the aid of high-performance next-generation sequencers, has allowed a better understanding of the gastric microbiota composition, as discussed in the next paragraphs [20].

Historically, the discovery of *Helicobacter pilory* demonstrated that the stomach was not sterile environment, thus prompting further studies on the gastric microbiota [21]. Accordingly, there is strong evidence that *Bacillota*, *Pseudomonadota*, *Bacteroidota*, *Actinomycetota* and *Fusobacteriota* are the major phyla in the gastric mucosa, while *Streptococcus*, *Prevotella*, *Fusobacterium*, *Veillonella*, *Neisseria*, and *Haemophilus* represent the most prevalent genera [22,23,24]. Of note, in the gastric fluid, *Actinomycetota*, *Bacteroidota*, and *Bacillota* are present in lower numbers than in the gastric mucosa [25]. In this framework, it is worth noting that the gastric microbiota composition greatly varies according to the stage of *H.p.* infection, gut microbiota, diet, age, immune status, and geography [26].

*H.p.* is a Gram-negative, flagellated, curved-rod bacterium that can survive in the hostile gastric milieu. It reaches the gastric mucus layer, using flagella, and via chemotaxis in response to mucin, sodium bicarbonate, sodium chloride, and urea [27].

Especially, *H.p.-*derived urease is a critical enzyme since it catalyzes the hydrolysis of urea to carbon dioxide and ammonia, thus buffering acidity in the gastric milieu [28]. Moreover, urea is a pathogenic factor in that it triggers the NF-κB pathway with the release of pro-inflammatory cytokines, disruption of epithelial tight junctions, and platelet aggregation [29]. Furthermore, adhesion molecules as well as surface receptors of gastric cells promote the binding of *H.p.* to the host [30]. Strains of *H.p.* with high expression of the blood group antigen binding adhesin A adhere more tenaciously to the gastric mucosa, provoking duodenal ulcer and gastric adenocarcinoma [31]. This may be due to a condition of *H.p.-*mediated dysbiosis in the gastric milieu, which gives rise to chronic inflammation, and ultimately, to cancer [32]. According to the literature, *H.p.-*dependent dysbiosis may rely on the alteration of the gastric microbiota composition, with a change in acidity, proliferation of certain bacterial strains, and interference with host lifestyle and diet [33].

As far as pathogenic mechanisms are concerned, the relationship between *H. pylori* and oral microbiota implies three steps: coaggregation, symbiotic biofilm formation, and endosymbiosis. *Fusobacterium nucleatum* and *Porphyromonas* (*P*.) *gingivalis*, typical oral pathogens, can aggregate *H. pylori*, forming biofilms and promoting inflammation and resistance to antibiotics. Of note, *P. gingivalis* represents a pathogenic agent of periodontitis and its association with *H. pylori* further aggravates periodontal disease. Also, *Streptococcus mutans* produces a symbiotic biofilm with *H. pylori*, allowing its survival in the oral environment [34].

Variations in the gastric microbiota depend on the stage of *H.p.-*related disease. For instance, in non-atrophic gastritis and intestinal metaplasia, *Neisseria*, *Porphyromonas*, *Streptococcus sinensis*, and *Saccharibacteria* tend to decrease, while *Lachnospiraceae* and *Lactobacillus coleohominis* tend to increase [35]. In gastric cancer patients, using pyrosequencing methods, the number of *H.p.* decreased by 86% at the family level, while *Streptococcaceae* and *Bacilli* increased at the class level [36]. Oral bacteria, i.e., *Aggregatibacter*, *Alloprevotella*, and *Neisseria*, increased in patients with gastric cancer in comparison to the superficial gastritis group [37]. Using 16S rRNA gene sequencing, in cancer biopsies, *Prevotella* spp. and *Clostridium* spp. increased, whereas *H.p.*, *Propionibacterium* spp., *Staphylococcus* spp., and *Corynebacterium* spp. decreased in comparison with non-cancer biopsies [38,39].

*H.p.* infection creates unfavorable conditions for other bacteria in the gastric habitat, altering luminal pH and producing a lot of molecules capable of inhibiting their normal replicative process. In fact, *H.p.*+ patients exhibited lower numbers of *Actinomycetota*, *Bacillota*, and *Bacteroidota*, while *Pseudomonadota* increased [40]. In animal studies, prolonged colonization of *H.p.* influenced the reproduction of *Lactobacillaceae* family, while *Bacteroides*, *Bifidobacteria*, *Staphylococcus aureus*, and *Enterococci* could adapt to the stomach environment [41].

The interference of *H. pylori* with *Lactobacillaceae* has been intensively investigated. The results are quite controversial, since *Lactobacillaceae* increased in *H. pylori*-infected persons in comparison to non-infected ones. The same authors reported reduced amounts of *Lactobacillus* (*L.*) *acidophilus* in *H. pylori*-positive individuals, but an increased proportion of *Ligilactobacillus salivarius* in comparison with non-infected subjects. Furthermore, in *H. pylori*-positive patients with atrophic gastritis compared to infected patients with mild atrophic gastritis or without gastritis, the abundance of *Lactobacillaceae* was more elevated. This suggests that the severity of *H. pylori* infection may influence the gastric colonization [42].

As also discussed in the next paragraphs, the interference of *H.p.* with non-*H.p.* bacteria may be involved in the development of chronic gastritis and cancer.

**Figure 1:**
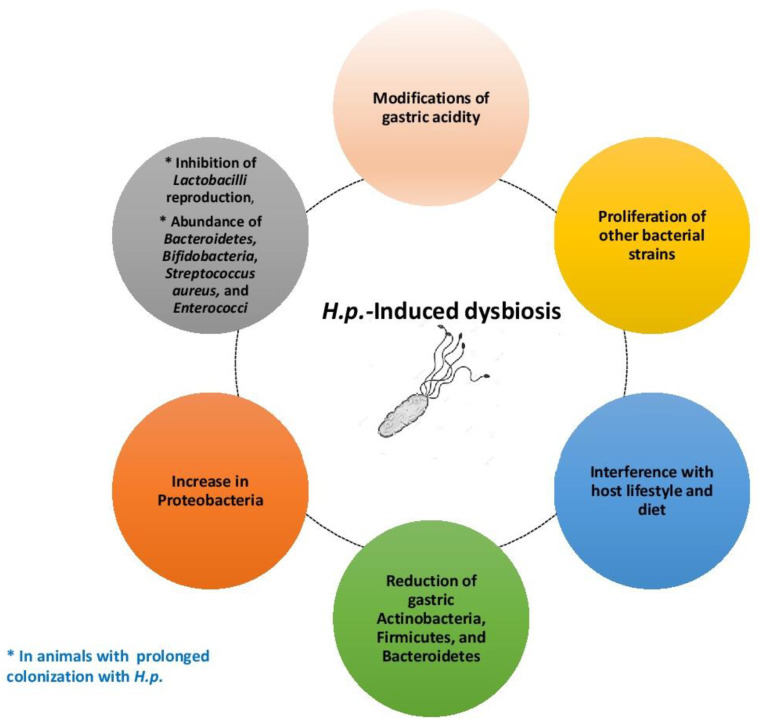
Mechanisms of dysbiosis provoked by *H.p.* infection.

In Figure 1, *H.p.-*induced dysbiosis at the gastric level is presented.

## 3. *H.p.-*Mediated Gastric Inflammation

*H.p.* is endowed with two major virulence factors, CagA and VacA. CagA is delivered into gastric epithelial cells by T4SS and is involved in carcinogenesis. CagA and VacA are virulence factors, with the former associated with gastric adenocarcinoma and the latter mostly associated with NF-kB-mediated inflammatory response [43].

Furthermore, CagA interacts with the hepatocyte growth factor receptor Met, thus triggering beta-catenin and NF-κB signaling, ultimately leading to gastric epithelial proliferation and inflammation. The penetration of CagA into the epithelium induces the secretion of interleukin (IL)-1 beta and IL-8, with the former initiating the inflammatory reaction and the latter chemotactically attracting neutrophils to the site of infection [44]. Cag A also triggers the release of IL-10, which, in turn, induces NF-κB activation and IL-8 expression, promoting the growth of bacteria. On the other hand, Vac A, once internalized into gastric mucosal cells, leads to a series of effects, such as cell vacuolization, mitochondrial stress, autophagy, suppression of T cell activity, and apoptosis [45,46].

**Figure 2:**
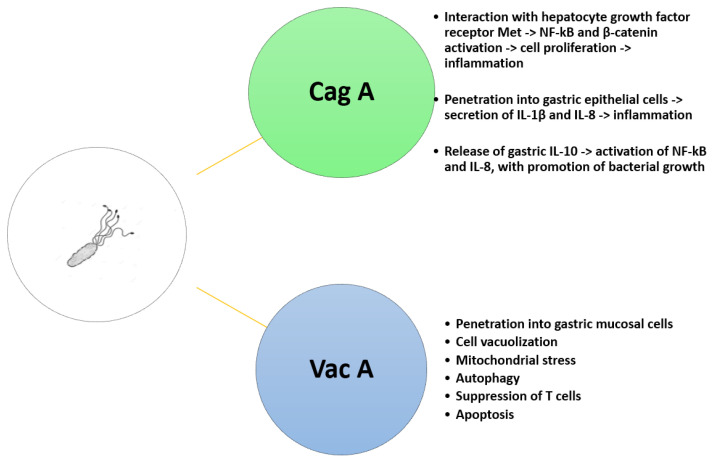
Pathological effects determined by Cag A and Vac A on gastric mucosa.

In Figure 2, the pathological effects elicited by CagA and VacA are summarized.

Quite importantly, the Vac-s1m1 genotype with high vacuolating activity is more expressed in chronic gastritis patients, while the Vac i1 genotype induces a higher expression of CD55 and contributes to the generation of the pro-inflammatory cytokine IL-8 [47,48].

Other inflammatory aspects during *H.p.* infection are represented by a reduced production of matrix-metallo-proteinase 7 with the activation of pro-inflammatory macrophages, M1, which perpetuate inflammation [49]. In fact, the infiltration of the gastric mucosa with macrophages and neutrophils triggers the release of another wave of pro-inflammatory cytokines, reactive oxygen species, and reactive nitrogen species [50,51]. During *H.p.* infection, the generation of cyclooxygenase (COX)-2 promotes the synthesis of prostaglandin (PG)E2, with the induction of the chemokine C-C motif ligand 2 [52].

Moreover, elevated PGE2 levels have been reported in the gastric juice of chronic gastritis patients [53]. It is worth noting that an amplification mechanism of *H.p.*-mediated inflammation has been documented through activated NF-κB, which mediates COX-2 expression and enhances levels of PGE2 and IL-8 in gastric epithelial cells [54,55,56].

Toll-like receptors (TLRs) are preferential targets for *H.p.* ligands. TLRs are type 1 transmembrane glycosylated proteins, which can detect microbes and/or their toxic products outside or inside cells [57]. *H.p.* interacts with TLR1, TLR2, TLR4, TLR5, and TLR10 on the cell surface, and with TLR9 inside the cell. TLR2 and TLR4 are ligands for *H.p.* LPS, while Cag A and flagellin interact with TLR5 [58,59]. TLR2 involvement gives rise to IL-8 overexpression, while TLR4 binding participates in the release of IL-1 beta and IL-12, which, in turn, induce the differentiation of pro-inflammatory subsets of T cells, namely Th1 and Th17 [60]. Such an inflammatory loop is aggravated by the reduced function of T regulatory (T_Reg_) cells in view of *H.p.*-mediated gastric microbiota dysbiosis [61]. In this respect, it is well known that T_Reg_ cells via the release of IL-10 suppress the Th1/Th 17 function [62]. Finally, TLR9 expression is increased in response to *H.p.* DNA, contributing to the gastric atrophy process [63].

In this framework, *H.p.* LPS deserves a special mention. In fact, it is less toxic than conventional LPS, and its activity, measured as endotoxin units, is 100-fold lower than that of *Escherichia* (*E.*) *coli* LPS [64,65]. *H.p.* LPS binds to TLR2, TLR4, and TLR5, initiating the inflammatory process of the gastric mucosa [66,67,68,69]. With special reference to *H.p.*-mediated TLR4 binding, this event leads to the proliferation of gastric epithelial cells via stimulation of the myeloid differentiation factor 2 (MD-2), with the increase in the activation of NF-κB and IL-8 promoters [70]. In gastric cancer, the higher expression of the TLR4/MD-2 pathway predicts a worse prognosis and poorer survival [71]. Moreover, the lower toxicity of *H.p.* LPS promotes tumor growth, decreasing the cytotoxic activity of mononuclear cells and natural killer (NK) cells [72], also contributing to immune evasion mechanisms [73,74].

**Figure 3:**
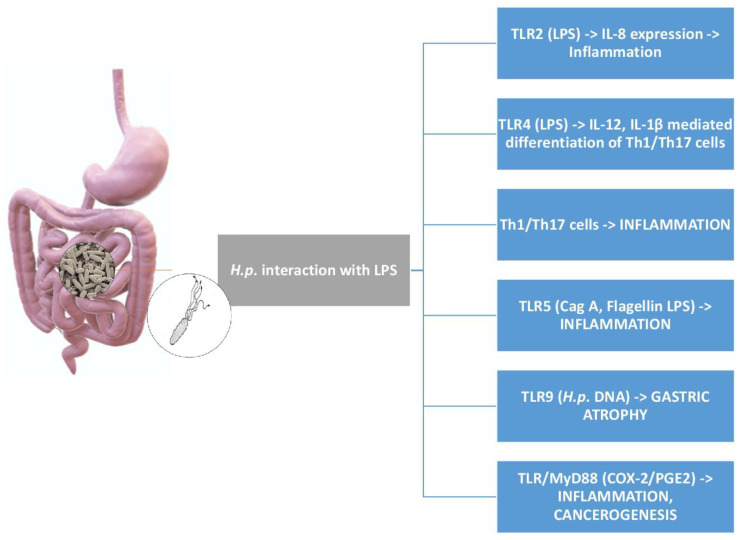
The network of interactions between *H.p.* and TLRs. TLR2, TLR4, TLR5, and TLR9 are ligands for different components of *H.p.*, leading to inflammation, gastric atrophy, and gastric cancer. The interplay between TLR/MyD88 and COX-2/PGE2 is involved in the inflammatory process and tumorigenesis.

Figure 3 illustrates the *H.p.* components involved in TLR-mediated gastric inflammation.

## 4. *H.p.* and Non-*H.p.* Organism-Mediated Gastric Tumorigenesis

CagA is an oncogenic protein which interacts with different signaling pathways. Phosphorylated Cag A interacts with activated SHP2, thus enhancing the Erk-MAP kinase signaling and ultimately leading to morphological changes (hummingbird phenotype) [75,76]. On the other hand, the non-phosphorylated CagA induces nuclear B-catenin accumulation, with the transcription of pro-carcinogenic genes. Furthermore, other cofactors are involved in *H.p.*-dependent gastric carcinogenesis, such as adhesins [BabA, SabA), outer membrane proteins (OipA, HomB), VacA, and proinflammatory cytokines [77,78].

Of note, the subversion of gastric mucosa by *H.p.* may be associated with the development of non-gastric tumor pathology, i.e., MALToma and gastrinoma. With special reference to MALToma, it is caused by the *H.p*. stimulation of mucosal-associated lymphoid tissue (rarely a B lymphoma) and disappears after *H.p.* eradication [79].

Non-*H.p.* bacteria can be associated with gastric cancer. *Lactobacillus* has been detected in the microbiota of gastric cancer [79]. This bacterium, a typical probiotic, produces lactic acid, which serves as an energy source for cancer cells, also triggering neo-angiogenesis. On the other hand, cell-free extracts of probiotics and supernatants of their fermentation reduce the genotoxicity of human fecal slurry, mainly thanks to butyric acid that is capable of enhancing the production of GST-pi in colon cells and binding irreversibly to mutagens, so inhibiting the genotoxicity of nitrosamides and H_2_O_2_ in colon cells [80]. *E. coli* is also present in the microbiota of gastric cancer and may exert specific genotoxicity by analogy to certain *E. coli* strains associated with colorectal cancer [81]. In the gastric microbiota, the abundance of nitrosating bacteria increases the intragastric concentration of nitrite and N-nitroso compounds, thus contributing to gastric cancer development [82]. *Fusobacterium* (*F.*) *nucleatum* has been detected in gastric cancer tissue, being associated with cancer risk, age of the patient, tumor size, and decreased survival [83,84,85]. It has been hypothesized that *F. nucleatum* may deregulate actin dynamics and cancer cell motility in the late phase of carcinogenesis.

Also, viruses have been implicated in gastric carcinogenesis. Epstein–Barr virus (EBV) participates in the early phase of gastric cancer development by promoting chronic inflammation [86]. Plasma EBV load is a good marker of recurrence and chemosensitivity and decreases after gastrectomy and chemotherapy [87]. Furthermore, EBV-noncoding RNA downregulate the miR-200 family with a reduction in E-cadherin expression and promotion of carcinogenesis.

*Candida albicans* is elevated in gastric cancer, but further studies are required to confirm its carcinogenic activity [88].

Table 1 describes the mechanisms of carcinogenesis mediated by *H.p.* and non-*H.p.* organisms.

## 5. The Relationship between Gastric Innate Lymphoid Cells and *H.p.*

The gastric mucosa contains a few lymphoid cells (ILCs), but under pathological circumstances, ILCs have mostly been demonstrated to maintain mucosal homeostasis, playing a crucial role in local immune responses [89]. ILCs have been divided into three main groups, ILC1s, ILC2s, and ILC3s [90,91,92]. ILC1s consist of NK cells and the helper-like ILC1 subset and produce interferon (IFN)-gamma upon activation [93]. ILC2s are characterized by the secretion of T helper (Th)2-related cytokines, such as IL-5 and IL-13 [94]. Finally, ILC3s consist of various subsets, e.g., CCR6-expressing lymphoid tissue inducer (LTi), natural cytotoxicity receptor (NCR)+, and NCR-ILC3s [95]. During gastric inflammation and tumorigenesis mediated by *H.p.*, ILCs play a fundamental role locally. NK cells, when activated by *H.p.*, proliferate and increase their antibacterial cytotoxicity, secreting IFN-gamma [96]. Such a process is potentiated by IL-12 produced by gastric macrophages and dendritic cells under *H.p.* stimulation [97]. Quite importantly, *H.p.* adopts escape mechanisms from the NK cell response, inhibiting IFN-gamma, IL-12, and perforin production [98,99]. Furthermore, the HopQ outer membrane protein of *H.p.* and the cecropin-like *H.p.* peptide inhibit NK cell function, also promoting apoptosis [100,101,102].

The role of ILC2s during *H.p.* infection is controversial. In fact, *H.p.* enhances the type 2 immunity mediated by ILC2s, with a decreased Th1 response [103]. Moreover, the *H.p.*-mediated upregulation of GATA-3 expression, a signature marker of ILC2s, reduces connexin 43, a major constituent of GAP junctions, and this may contribute to gastric cancer development [104]. Furthermore, IL-33 produced by the *H.p.-*infected gastric mucosa plays either a protective or a harmful role. In the early stage of infection, IL-33 activates ILC2s, which, in turn, prevent Th1/Th17-mediated inflammation [105,106].

Conversely, in the late stage of infection, IL-33 decreases, leading to M2 macrophage polarization, with the increase in STAT3 activation and promotion of tumor growth. Finally, ILC2s activate B cells into IgA-producing plasma cells, which are protective against *H.p.* at the mucosal level [107].

**Figure 4:**
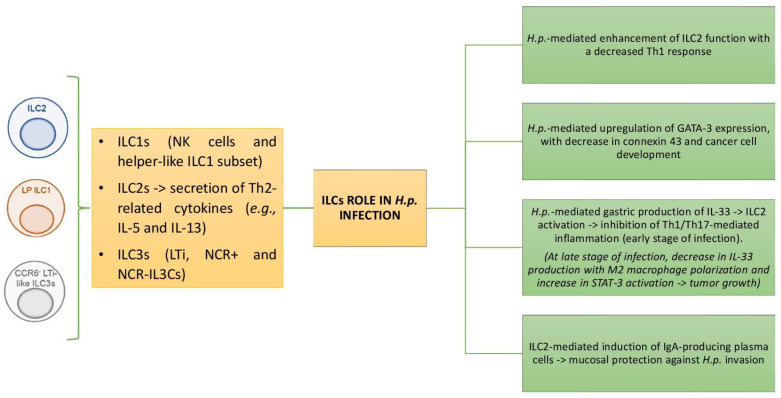
Role of innate lymphoid cells during *H.p.* infection.

Figure 4 illustrates the relationship between ILC2s and *H.p.* and the effects at the gastric mucosa level.

## 6. Use of Natural Products to Treat *H.p.* Infection

Different regimens are currently used for *H.p.* eradication, and, among them, proton pump inhibitors (PPIs), triple therapy, bismuth-containing quadruple therapy, modified regimens, concomitant therapy, hybrid therapy, and sequential therapy are the most common [108].

However, PPIs and H2 blockers are used to contrast *H.p.*-mediated pathologies. However, the putative risk to provoke gastric cancer for periods beyond 3 months should be taken into serious consideration.

It is well known that *H.p.* eradication influences gastric microbial composition and function, but whether bacterium eradication normalizes the gastric microbiota to an uninfected status is still a matter of debate [109]. However, there are good lines of evidence that suggest the beneficial effects of natural product administration during *H.p.-*mediated infections.

### 6.1. Effects of Probiotics and Prebiotics on the Gastric Microbiota

Probiotics are living microorganisms which exert protective effects on the host when supplemented in adequate amounts [110], and, for instance, evidence has been provided that probiotic administration attenuates gastric inflammation [111,112]. In fact, in animal models infected with *H.p.*, different strains of *Lactobacillaceae* could inhibit NF-κB activity, with a decrease in the release of pro-inflammatory cytokines, *i.e.*, IL-1 beta, IL-8, Tumor Necrosis Factor(TNF)-alpha, and IFN-gamma, as well as *H.p.-*specific IgM and IgA levels in the stomach [113,114]. In this scenario, it has been suggested that the activation of dendritic cells by probiotics, as well as lactic acid production, may contribute to reducing *H.p.-*mediated inflammation [115,116]. Furthermore, probiotics can lead to *H.p.* eradication [117]. In this respect, their administration together with standard antibiotic treatment enhanced *H.p.* eradication, also reducing antibiotic-related adverse effects [118,119,120]. Moreover, *Limosilactobacillus reuteri* through the secretion of reuterin, a potent antimicrobial agent, could inhibit *CagA* and *VacA* genes [121]. Also, *H.p.* metabolites, such as polysaccharides, surface and secreted proteins, peroxide, indole, and extracellular vesicles, induced mucous secretion in goblet cells, affording protection against *H.p.* [122,123].

Prebiotics are substrates used by host-health-promoting microorganisms [124]. They consist of inulin, oligofructose, lactulose, resistant starch, and wheat bran [125]. Prebiotics promote the growth of *Bifidobacterium* species and *Lactobacillaceae* genera in the gut, with the production of short-chain fatty acids (SCFAs) and improvement in gut barrier function and mineral absorption [126]. The co-administration of probiotic strains with fructooligosaccharides (FOSs) as a prebiotic (synbiotic) together with the conventional anti-*H.p.* regimen could increase the rate of *H.p.* eradication [127]. Fucoidan, administered to *H.p.+* patients together with the standard quadruple therapy (SQT), could mitigate symptoms in eradicating *H.p.*, improving SQT-dependent dysbiosis [128].

Table 2 reports the effects of probiotics and prebiotics on *H.p.* infection.

### 6.2. Effects of Polyphenols on the Gastric Microbiota

Polyphenols are largely distributed in the vegetal kingdom, and fruits, vegetables, grains, soy, tea, wine, and extra virgin olive oil are major sources [129]. There is evidence that a diet enriched in polyphenols protect against chronic disease, in view of their antioxidant and anti-inflammatory effects [130,131]. Quite interestingly, polyphenols afford protection against *H.p.* infection, also preventing gastric cancer risk factors. With special reference to anti-*H.p.* activity, it has been reported that resveratrol, red wine, and procyanidins inhibit *H.p.* urease [132,133,134]. Furthermore, flavonoids decrease the adhesion between *H.p.* and the gastric mucosa, thus leading to 90% of *H.p.* growth inhibition [135]. Also, epicatechin reduced the adhesion of *H.p.* to human gastric cancer cells, thus inhibiting IL-8 production [136,137,138]. Further research demonstrated the ability of polyphenols to decrease the translocation of CagA and VacA. In fact, kaempferol, catechin, resveratrol, and curcumin could inhibit either in vitro or in vivo Vac A-induced vacuolization, mRNA, and protein levels of CagA and VacA, limiting the damage of the gastric epithelium [139,140,141].

There is evidence that polyphenols can play a protective role against *H.p.-*mediated gastric cancer. In fact, curcumin inhibits gastric cancer growth via the production of reactive oxygen species, with the depletion of mitochondrial DNA content and DNA polymerase, thus leading to the repair of damaged DNA [142]. Moreover, curcumin analogs are inhibitors of topoisomerase II, thus causing cancer cell apoptosis [143]. Also, curcumin is endowed with apoptotic ability by the Wnt/catenin pathway activation and increase in the expression of Bcl-2 and Bcl-xL [144,145]. Resveratrol has been reported to arrest gastric cancer cell proliferation and survival, hampering PIM-1 kinase activity [146]. Among other activities, polyphenols prevent the epithelial–mesenchymal transition during gastric cancer, inhibiting the hedgehog and PTEN/Akt pathways, respectively [147,148]. In this framework, it has been documented that polyphenols can inhibit gastric cancer metastasis via the inhibition of IL-6, MM9/2 expression, and the CD1/CXR4 and HMGB1/VEGF-D pathways [149,150,151,152].

Despite the evidence that *H.p.* is a pathogenic factor in gastric cancer, this bacterium can enhance PD-L1 expression in primary human gastric epithelial cells, as well as reinforce the efficacy of the therapy with checkpoint inhibitors [153].

Conclusively, a combination of polyphenols is more effective than single preparations, and, therefore, more studies are needed to determine optimal combinations.

**Figure 5:**
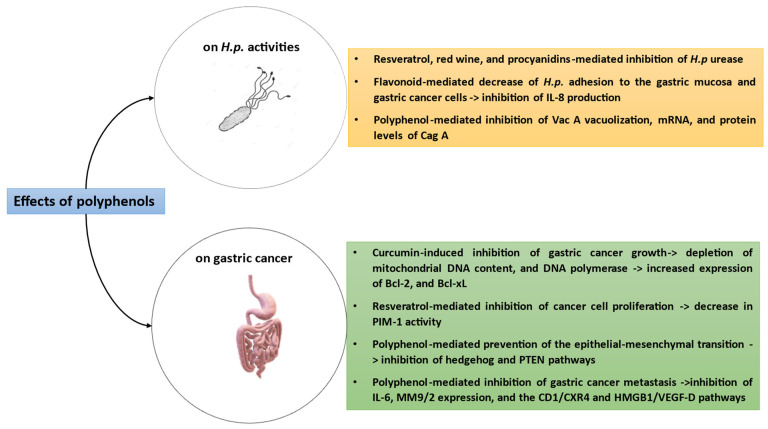
Various mechanisms exerted by polyphenols on *H.p.* activity, even including protective effects against gastric cancer.

Figure 5 elucidates the protective mechanisms exerted by polyphenols during *H.p.-* mediated pathologies.

In this context, it is worth mentioning that the microbiota and their metabolites may contribute to gastric cancer therapy, promoting the secretion of cytokines and T cytolytic cell infiltration [154,155]. Moreover, fecal microbiota transplantation (FMT) in *H.p.-*infected patients is associated with a significant eradication of this bacterium [156]. Experimentally, FMT improved the efficacy of checkpoint inhibitor therapy, currently used in cancer cell patients [157]. Therefore, FMT in gastric cancer patients may have large prospects.

Finally, the use of nanoparticles is noteworthy for the treatment of *H.p.* infection, proposed to overcome emerging antimicrobial resistance [158,159]. In one of the more recent studies, chitosan nanoparticles conjugated with antimicrobial peptides have been used against antibiotic-resistant *H.p.* strains, leading to an effective bacterial destruction [160].

## 7. Conclusions

*H. pylori* infection is very frequent in developing countries and eradication therapy is recommended to avoid a cancer outcome. However, eradication is complicated by the necessity to use both antibiotics and PPIs. Despite these remedies, the risk of antibiotic resistance is elevated, with high cost on the national level, and the development of microbiota dysbiosis. With special reference to therapeutic approaches, the major goals in *H. pylori*-infected individuals are the correction of gut microbiota, the enhancement in humoral and cellular immunity, and the reduction in oxidative stress in the gastric milieu. However, one should consider possible side-effects of alternative treatments of *H. pylori*-infected patients, even in the case of probiotics, which include systemic infections, long-term gut dysbiosis, and risks of developing Parkinson’s disease.

There is clear-cut evidence that *H.p.* and non-*H.p.* bacteria are involved in gastric inflammation and gastric cancer outcomes. With special reference to gastric cancer progression, the mechanism by which gastric microbiota promotes tumorigenesis needs further investigation. To accomplish this goal, novel sequencing procedures may clarify the most predominant bacteria in gastric carcinoma tissue, gastric mucosa, and fecal samples, on the one hand. On the other hand, broad prospective cohort studies may help in understanding the gastric-microbiota-mediated tumorigenesis, and its modifications during the long-term progression of gastric cancer.

Supplementation with natural products, i.e., probiotics, prebiotics, and polyphenols, have been demonstrated to provide beneficial effects during *H.p.-*related pathologies, mostly in combination with the conventional anti-*H.p.* therapeutic regimen. Finally, FMT combined with immunotherapy may improve the therapeutic effects and survival rate in gastric cancer patients.

## Figures and Tables

**Table 1:** *H.p.* and non-*H.p.-*mediated gastric cancer development. Various mechanisms of carcinogenesis are described.

Mechanisms of Gastric Cancer Development	
*H.p.*-Mediated	Enhancement in the Erk-MAP kinase due to CagA interaction with activated SHP2; Accumulation of nuclear β-catenin and transcription of pro-carcinogenic genes exerted by non-phosphorylated CagA; Other factors: adhesins (BabA, SabA), membrane proteins (OipA, HomB), Vac A, proinflammatory cytokines.
Non-*H.p.*-Mediated	*Lactobacillus*-dependent production of lactic acid as energy source for cancer cells and promotion of neo-angiogenesis; *E. coli* increased genotoxicity; Abundance of nitrosating bacteria with higher concentration of intragastric nitrite and N-nitroso compounds; *F. nucleatum*-mediated deregulation of actin dynamics and cancer cell motility; EBV-noncoding-RNA-mediated downregulation of miR-200 family with reduction in E-cadherin expression.

**Table 2:** Effects on *H.p.* infection-mediated by administration of probiotics and prebiotics.

Major Effects of Probiotics and Prebiotics in *H.p.* Infection	
Probiotics	Prebiotics
In experimental models, lactobacilli-mediated inhibition of NF-κB, pro-inflammatory cytokines, and *H.p*.-specific gastric IgM and IgA antibodies Anti-*H.p*.-mediated inflammatory activity by various strains belonging to *Lactobacillaceae* family Inhibition of *CagA* and *VacA* genes by *L. reuteri* Enhancement of goblet cell secretion by *H.p.* metabolites (polysaccharides, surface and secreted proteins, peroxide, indole, and extracellular vesicles)	Increase in the rate of *H.p.* eradication following co-administration of probiotics with prebiotics (synbiotic) together with the conventional anti-*H.p*. antibiotic regimen Fucoidan-induced symptom mitigation, and *H.p*. eradication in patients treated with SQT

## Data Availability

All available data have been reported in the manuscript.

## Abbreviations

Cag A	Cytotoxin-associated gene A
COX	cyclooxygenase
EBV	Epstein–Barr Virus
FMT	Fecal microbial transplantation
FOS	Fructooligosaccharides
*H.p.*	*Helicobacter pylori*
IFN	Interferon
IL	Interleukin
ILCs	Innate lymphoid cells
LPS	Lipopolysaccharides
MD-2	Myeloid differentiation factor-2
NCR	Natural cytotoxic receptor
NK	Natural killer
PG	Prostaglandin
SQT	Standard quadruple therapy
T4SS	Type IV secretion system
Th	T helper
TLRs	Toll-like receptors
Vac A	vacuolar toxin A
